# Supplementary material for: Wildfires enhance phytoplankton production in tropical oceans
Source: Nat Commun. 2022 Mar 15;13:1348. doi: 10.1038/s41467-022-29013-0 (PMC8924273; doi:10.1038/s41467-022-29013-0)
Supplement: Supplementary file 1 — Supplementary Information [file 41467_2022_29013_MOESM1_ESM.pdf]

Supplementary Information to:

Wildfires enhance phytoplankton production in tropical oceans

Dongyan Liu[1, *], Chongran Zhou[1], John K. Keesing[2, *], Oscar Serrano[3, 4], Axel Werner[3], Yin Fang[5], Yingjun Chen[6, *], Pere Masque[3, 7, 8], Janine Kinloch[9], Aleksey Sadekov[10], Yan Du[11]

[1] State Key Laboratory of Estuarine and Coastal Research, Institute of Eco-Chongming, East China Normal University, Shanghai 200062, China.
[2] CSIRO Oceans and Atmosphere Research, and University of Western Australia Oceans Institute, Indian Ocean Marine Research Centre, Crawley, WA, Australia.
[3] School of Science and Centre for Marine Ecosystems Research, Edith Cowan University, Joondalup, Australia.
[4] Centro de Estudios Avanzados de Blanes, Consejo Superior de Investigaciones Científicas, Blanes, Spain.
[5] College of Marine Ecology and Environment, Shanghai Ocean University, Shanghai 201306, China.
[6] Shanghai Key Laboratory of Atmospheric Particle Pollution and Prevention, Department of Environmental Science and Engineering, Fudan University, Shanghai 200438, China.
[7] Departament de Física and Institut de Ciència i Tecnologia Ambientals, Universitat Autònoma de Barcelona, Bellaterra, Spain.
[8] International Atomic Energy Agency, 4a Quai Antoine 1er, 98000 Principality of Monaco, Monaco.
[9] Biodiversity and Conservation Science, Department of Biodiversity, Conservation and Attractions, Bentley Delivery Centre, WA, Australia.
[10] Ocean Graduate School, ARC Centre of Excellence for Coral Reef Studies, University of Western Australia, Crawley, 6009, Australia.
[11] State Key Laboratory of Tropical Oceanography, South China Sea Institute of Oceanology, Chinese Academy of Sciences, Guangzhou, China

Correspondence to:

D. Liu, J. K. Keesing, and Y. Chen.

dyliu@sklec.ecnu.edu.cn; john.keesing@csiro.au; and yjchenfd@fudan.edu.cn

Supplementary Figure 1. **The variation of Char/Soot ratios in the three sediment cores**. **a** Core 185. **b** Core 200. **C** core KGR.

Supplementary Figure 2. **Profiles of grain sizes in the three cores, including the median grain size (d_{50}) and proportions of clay (below 4 µm), silt (4-63 µm), and sand (above 63 µm). a-b** core 185. **c-d** core 200. **e-f** core KGR.

Supplementary Figure 3. **The variation of sea surface temperature (SST), tropical cyclones frequency, and rainfall from 1920 to 2017** (blue lines represent the shift changes assessed by sequential t test analysis of regime shift) and the numbers were regime shift index). **a** SST. **b** tropical cyclone frequency. **c** rainfall.

Supplementary Figure 4 **The variation of $^{210}Pb_{ex}$ (±standard error of mean) in the three sediment cores**. **a** core 185. **b** core 200. **c** core KGR.

Supplementary Figure 5. **The variation of iron and potassium in core 200** (Blue lines represent the shift changes assessed by sequential t test analysis of regime shift and numbers on lines were regime shift index (RSI) to show shifting magnitude, and *r* and *p* values represent the Pearson correlations between BC and the two proxies, n=23).

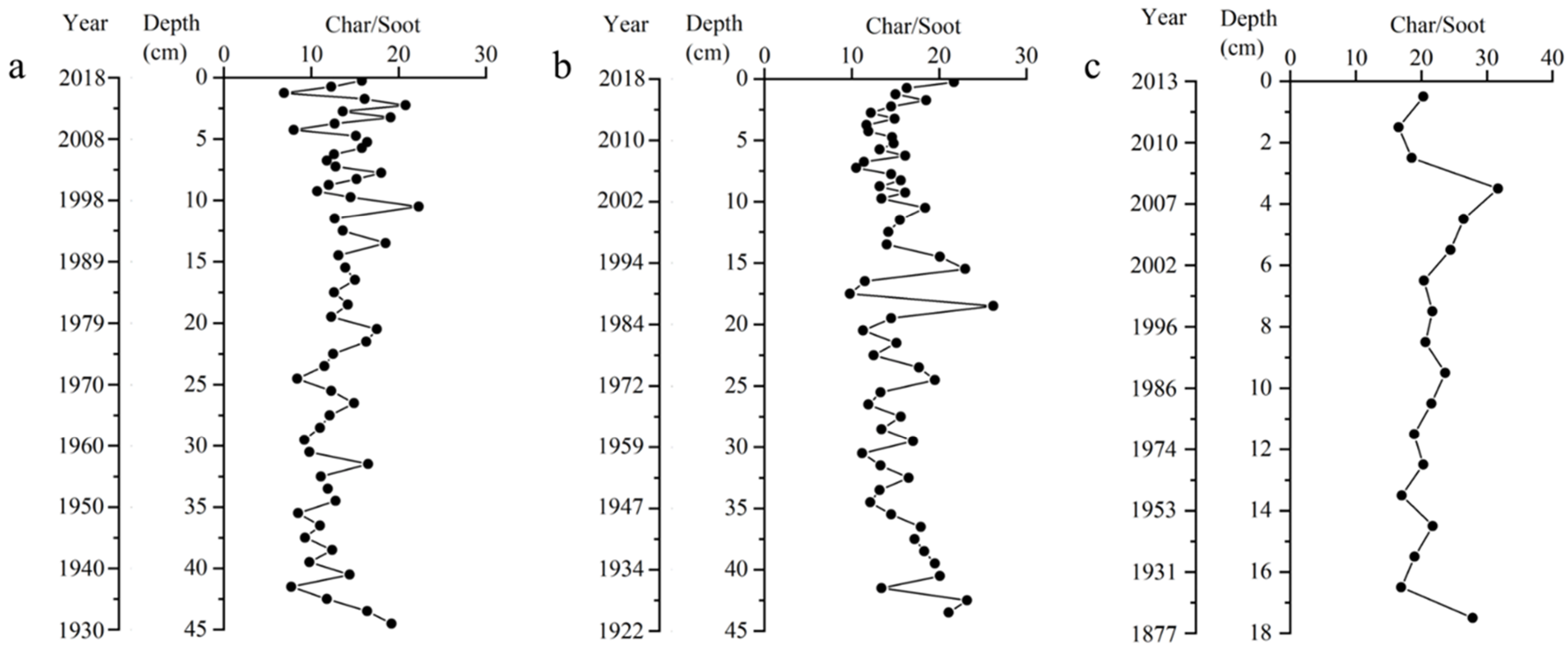

Supplementary Figure 1

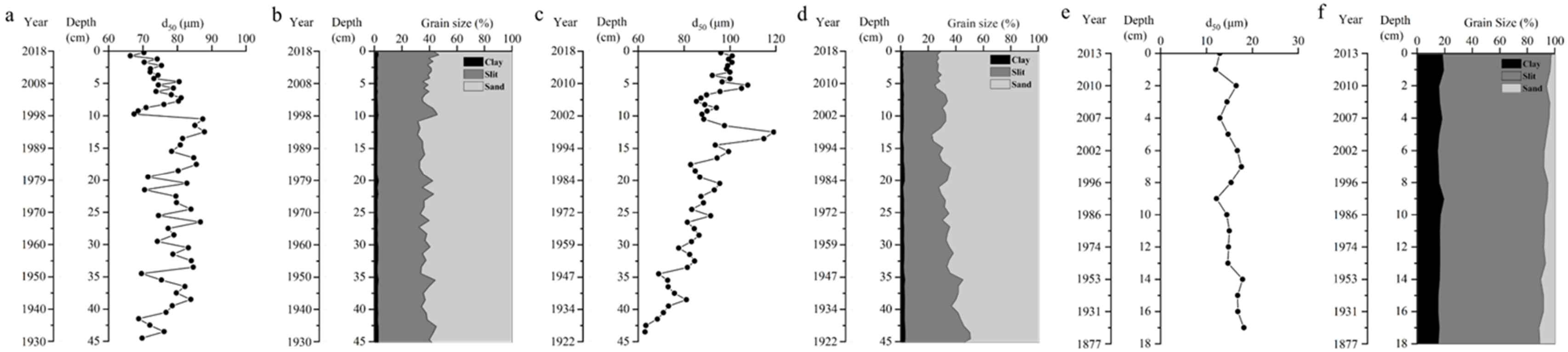

Supplementary Figure 2

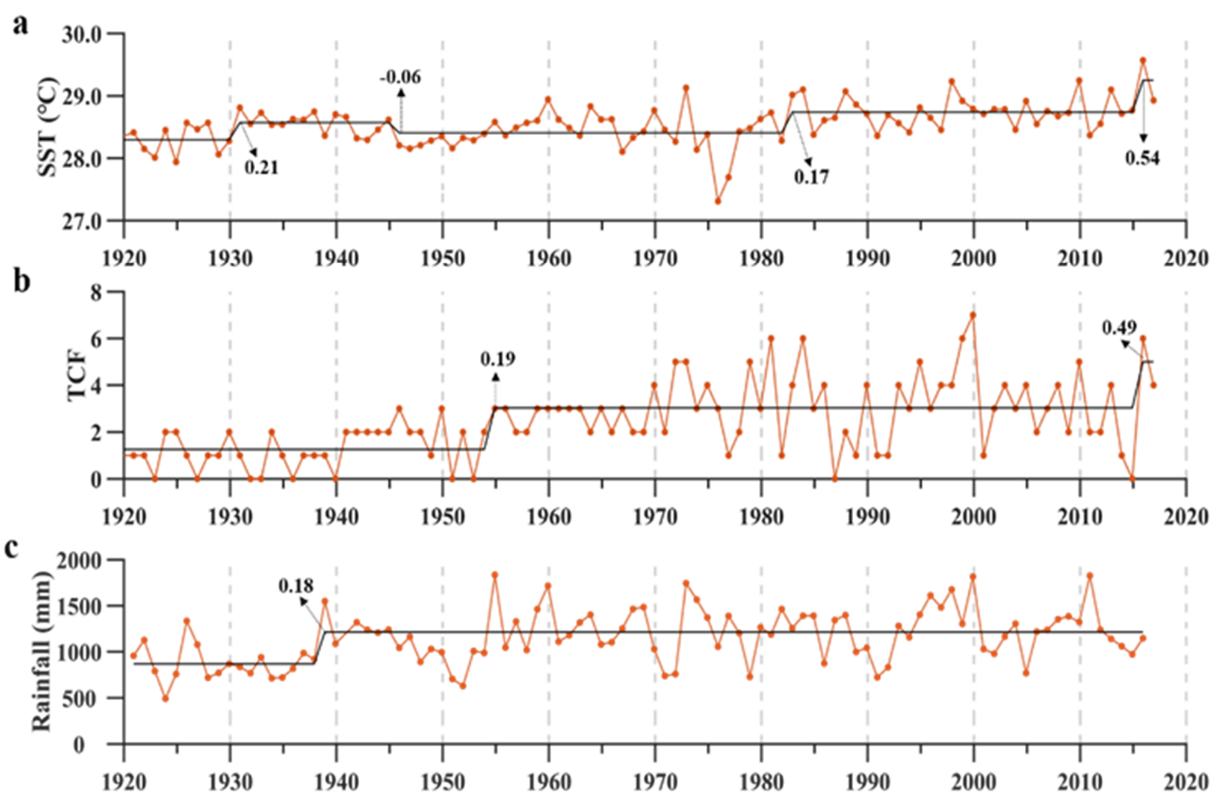

Supplementary Figure 3

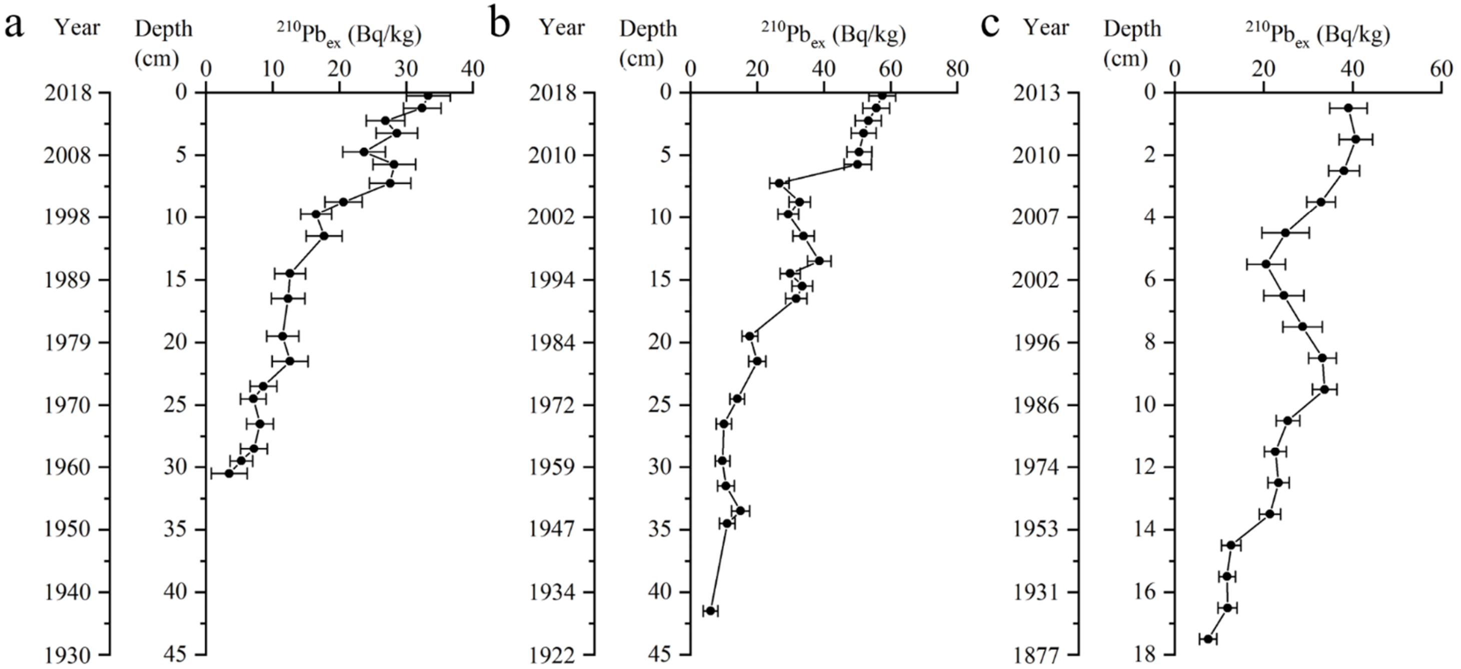

Supplementary Figure 4

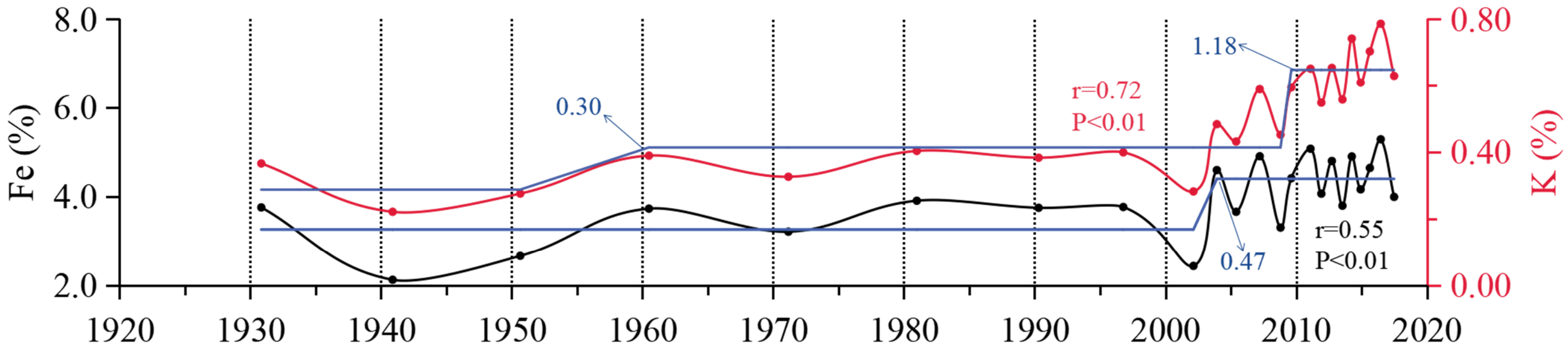

Supplementary Figure 5
