# Supplementary material for: Wildfires enhance phytoplankton production in tropical oceans
Source: Nat Commun. 2022 Mar 15;13:1348. doi: 10.1038/s41467-022-29013-0 (PMC8924273; doi:10.1038/s41467-022-29013-0)
Supplement: Supplementary file 3 — Reporting Summary [file 41467_2022_29013_MOESM3_ESM.pdf]

nature portfolio

Corresponding author(s): Dongyan Liu

Last updated by author(s): Jan 17, 2022

Reporting Summary

Nature Portfolio wishes to improve the reproducibility of the work that we publish. This form provides structure for consistency and transparency in reporting. For further information on Nature Portfolio policies, see our Editorial Policies and the Editorial Policy Checklist.

Statistics

For all statistical analyses, confirm that the following items are present in the figure legend, table legend, main text, or Methods section.

n/a	Confirmed	
☐	☒	The exact sample size (*n*) for each experimental group/condition, given as a discrete number and unit of measurement
☐	☒	A statement on whether measurements were taken from distinct samples or whether the same sample was measured repeatedly
☐	☒	The statistical test(s) used AND whether they are one- or two-sided<br>*Only common tests should be described solely by name; describe more complex techniques in the Methods section.*
☐	☒	A description of all covariates tested
☐	☒	A description of any assumptions or corrections, such as tests of normality and adjustment for multiple comparisons
☐	☒	A full description of the statistical parameters including central tendency (e.g. means) or other basic estimates (e.g. regression coefficient) AND variation (e.g. standard deviation) or associated estimates of uncertainty (e.g. confidence intervals)
☐	☒	For null hypothesis testing, the test statistic (e.g. *F*, *t*, *r*) with confidence intervals, effect sizes, degrees of freedom and *P* value noted<br>*Give P values as exact values whenever suitable.*
☒	☐	For Bayesian analysis, information on the choice of priors and Markov chain Monte Carlo settings
☒	☐	For hierarchical and complex designs, identification of the appropriate level for tests and full reporting of outcomes
☐	☒	Estimates of effect sizes (e.g. Cohen's *d*, Pearson's *r*), indicating how they were calculated

Our web collection on statistics for biologists contains articles on many of the points above.

Software and code

Policy information about availability of computer code

Data collection	no software or code was used in data generation
Data analysis	The timing and magnitude of shift changes in the biogeochemical proxies was assessed using the sequential t-test analysis of regime shifts (STARS). The STARS algorithm converted to VBA for Excel is available from www.BeringClimate.noaa.gov.

For manuscripts utilizing custom algorithms or software that are central to the research but not yet described in published literature, software must be made available to editors and reviewers. We strongly encourage code deposition in a community repository (e.g. GitHub). See the Nature Portfolio guidelines for submitting code & software for further information.

Data

Policy information about availability of data

All manuscripts must include a data availability statement. This statement should provide the following information, where applicable:

- Accession codes, unique identifiers, or web links for publicly available datasets
- A description of any restrictions on data availability
- For clinical datasets or third party data, please ensure that the statement adheres to our policy

All data analyzed during this study are included in an MS Excel data file as supplementary information,and all open source data is given its source.

March 2021

Field-specific reporting

Please select the one below that is the best fit for your research. If you are not sure, read the appropriate sections before making your selection.

☐ Life sciences ☐ Behavioural & social sciences ☒ Ecological, evolutionary & environmental sciences

For a reference copy of the document with all sections, see nature.com/documents/nr-reporting-summary-flat.pdf

Ecological, evolutionary & environmental sciences study design

All studies must disclose on these points even when the disclosure is negative.

Study description	This study is based on three sediment cores of approximately 1.5 m in length collected with the northwestern coast of Australia, which were determined over a depth range of nearly 100 years. By determining the biogeochemical parameters of the two sediment cores, we found a very strong correspondence between BSi used to characterize diatom productivity and black carbon used to characterize fire intensity in recent decades, which is likely to be related to the chronological oscillations of the ENSO and IOD.
Research sample	The target samples used in this study are three sediment cores collected from Napier Broome Bay (NBB) and Koolama Bay (KB), Western Australia. There are two sediment cores from NBB (core 185 and core 200) taken off the mouths of the King Edward and the Drysdale Rivers respectively. Core 185 (131 cm) was located in 14°02'S, 126°35'E, and in-situ water depth is 20.2 m. Core 200 (136 cm) was located in 13°53'S, 126°45'E, and in-situ water depth is 13.7 m. Core KGR was taken from 13°55'S, 127°19'E in 11.8 m depth. To ensure a high enough resolution, the cores were sliced every 0.5 cm along the upper 10 cm and the rest part was sectioned at 1-cm intervals
Sampling strategy	Sediment cores were collected in Napier Broome Bay (May 2018) and Koolama Bay (June 2013) using a vibrating head corer (Specialty Devices, Texas, US). The cores were sliced every 0.5 cm along the upper 10 cm and the rest part was sectioned at 1!cm intervals. All samples were stored in a freezer at -20 °C before dating and chemical analysis.
Data collection	The concentration profile of 210Pb in the core was determined by measuring its granddaughter 210Po radioactive equilibrium using alpha spectrometry34 at Edith Cowan University, Australia. Grain sizes were measured using a Malvern Mastersizer 2000F Laser Particle Sizer. The TOC and TN contents in the sediment core were measured using an elemental analyser (FlashSmart NC Soil, Thermo Scientific). BSi measurements were performed using an INESA-L8 ultraviolet-visible spectrophotometer. Sedimentary BC was quantified using the wet-chemical pre-treatment integrated with the thermal optical reflectance (TOR) method. Potassium and iron were measured following EPA method 3052 (ICP-MS, and standard reference materials were NIST 2702 and MESS-3).
Timing and spatial scale	Core 185 (131 cm) was located at 14°02'S, 126°35'E, and in-situ water depth was 20.2 m. Core 200 (136 cm) was located in 13°53'S, 126°45'E, and in-situ water depth was 13.7 m. Both sediment cores were collected in May 2018, and based on the dating results, core 185's data segment used in this study can cover the years from 1929 to 2018, while core200's can cover the years from 1926 to 2018. Core KGR was taken from 13°55'S, 127°19'E in 11.8 m depth in June 2013 and covers the period 1880 to 2013.
Data exclusions	No data was excluded from the analyses.
Reproducibility	In this study, we used three sediment cores across three river systems to ensure a wide representative spatial coverage. In addition, we applied multiple biogeochemical indicators to ensure the reliability of the conclusions.
Randomization	The study design did not require randomization and no data points were excluded from the analyses.
Blinding	Blinding was not relevant to this study. There was no subjective scoring criteria or measurements used.

Did the study involve field work? ☒ Yes ☐ No

Field work, collection and transport

Field conditions	Fieldwork involved collecting the sediments cores from small ships in Koolama Bay and Napier Broome Bay,Western Australia.
Location	Northern Kimberley coast, Western Australia
Access & import/export	Collecting permit from the relevant authority was obtained. A portion of the split samples were taken from Australia to China for particle size, elemental, biogenic silica and black carbon determinations.
Disturbance	The cores disturb only a small area of the muddy seabed. No other disturbance was made

Reporting for specific materials, systems and methods

We require information from authors about some types of materials, experimental systems and methods used in many studies. Here, indicate whether each material, system or method listed is relevant to your study. If you are not sure if a list item applies to your research, read the appropriate section before selecting a response.

Materials & experimental systems

n/a	Involved in the study
☒	☐ Antibodies
☒	☐ Eukaryotic cell lines
☒	☐ Palaeontology and archaeology
☒	☐ Animals and other organisms
☒	☐ Human research participants
☒	☐ Clinical data
☒	☐ Dual use research of concern

Methods

n/a	Involved in the study
☒	☐ ChIP-seq
☒	☐ Flow cytometry
☒	☐ MRI-based neuroimaging
